# Pain and dyspnea control during awake fiberoptic bronchoscopy in critically ill patients: safety and efficacy of remifentanil target-controlled infusion

**DOI:** 10.1186/s13613-021-00832-6

**Published:** 2021-03-16

**Authors:** Margot Caron, Antoine Parrot, Alexandre Elabbadi, Sophie Dupeyrat, Matthieu Turpin, Thomas Baury, Sacha Rozencwajg, Clarisse Blayau, Jean-Pierre Fulgencio, Aude Gibelin, Pierre-Yves Blanchard, Séverine Rodriguez, Daisy Daigné, Marie-Cécile Allain, Muriel Fartoukh, Tài Pham

**Affiliations:** 1 Sorbonne Université, Assistance Publique – Hôpitaux de Paris, Service de médecine intensive réanimation, Hôpital Tenon, Paris, France; 2 Université Paris-Saclay, AP-HP, Service de médecine intensive-réanimation, Hôpital de Bicêtre, DMU CORREVE, FHU SEPSIS, Groupe de recherche clinique CARMAS, Le Kremlin-Bicêtre, France; 3 Groupe de Recherche Clinique GRC05 CARMAS, Institut Mondor de recherche biomédicale, INSERM, Créteil, France

**Keywords:** Flexible fiberoptic bronchoscopy, Remifentanil target-controlled infusion, Intensive care unit, Tolerance, Comfort

## Abstract

**Purpose:**

Flexible fiberoptic bronchoscopy is frequently used in intensive care unit, but is a source of discomfort, dyspnea and anxiety for patients. Our objective was to assess the feasibility and tolerance of a sedation using remifentanil target-controlled infusion, to perform fiberoptic bronchoscopy in awake ICU patients.

**Materials, patients and methods:**

This monocentric, prospective observational study was conducted in awake patients requiring fiberoptic bronchoscopy. In accordance with usual practices in our center, remifentanil target-controlled infusion was used under close monitoring and adapted to the patient’s reactions. The primary objective was the rate of successful procedures without additional analgesia or anesthesia. The secondary objectives were clinical tolerance and the comfort of patients (graded from “very uncomfortable” to “very comfortable”) and operators (numeric scale from 0 to 10) during the procedure.

**Results:**

From May 2014 to December 2015, 72 patients were included. Most of them (69%) were hypoxemic and admitted for acute respiratory failure. No additional medication was needed in 96% of the patients. No severe side-effects occurred. Seventy-eight percent of patients described the procedure as “comfortable or very comfortable”. Physicians rated their comfort with a median [IQR] score of 9 [8–10].

**Conclusion:**

Remifentanil target-controlled infusion administered to perform awake fiberoptic bronchoscopy in critically ill patients is feasible without requirement of additional analgesics or sedative drugs. Clinical tolerance as well as patients’ and operators’ comfort were good to excellent. This technique could benefit patients’ experience.

**Supplementary Information:**

The online version contains supplementary material available at 10.1186/s13613-021-00832-6.

## Background

Pain management remains a major challenge in intensive care units (ICU), and common ICU procedures, such as fiberoptic bronchoscopy (FOB) may induce important levels of pain [1–4]. The International Association for the Study of Pain has defined procedural pain as “the unpleasant sensory and emotional experience that arises from actual or potential tissue damage associated with diagnostic or treatment procedures” [5], of which an inadequate management may have major consequences. Under-treatment of pain in post-operative period generates specific complications: sympathetic response increases levels of circulating catecholamines leading to an elevation of heart rate, blood pressure, with the risk of myocardial ischemia or bleeding [6–8]. In critically ill patients, memory of pain is a major risk of post-traumatic stress syndrome, which may be persistent and impairs patient rehabilitation and family dynamics [9]. Unrelieved ache has been identified as one of the main source of psychological stress for ICU patients [10]. Patients in the ICU often experience several procedures, or the same procedure repeated several times, such as central line or arterial catheters insertion, diagnostic or therapeutic fiberoptic bronchoscopies. Each new procedure adds anxiety to an already stressful situation [11]. Because procedural pain can be anticipated, it can also be relieved [1, 2]. Under-treated, suffering has medical consequences, but also impairs the condition under which the examinations are performed, with major risk of patients’ agitation and failure of the procedure. Evaluating and treating pain is of paramount importance and is a simple means to decrease duration of mechanical ventilation and ICU length of stay by limiting the use of sedatives and neuromuscular blockers [12].

In many situations patients are unable to report or verbalize pain and discomfort due to intubation or altered consciousness [1]. Besides, variation of physiologic parameters, often used as an indicator of pain, is insufficient when used alone [13]. Behavioral response, well correlated with the intensity of procedural pain, seems to be a good option to enhance patient’s assessment [1, 13, 14]. Pain should be routinely monitored in ICU patients, and preemptive analgesia should be administered to alleviate pain before any painful action. The clinical practice guidelines for pain, agitation and delirium recommend the use of intravenous opioids in such circumstance [15].

Remifentanil is a potent, ultra-short selective µ opioid, with a short context-sensitive half-time, allowing prolonged infusion without excessive accumulation [16, 17]. It is hydrolyzed by plasmatic and tissues nonspecific esterase, and undergoes rapid metabolism, independent of renal and liver functions [18, 19]. This organ-independent metabolism is a very interesting characteristic for intensive care patients who often present with organ dysfunctions. Remifentanil has been used with a target-controlled infusion (TCI) pharmacokinetic model in non-intubated critically ill patients requiring FOB with a good tolerance [20]. A recent study showed that remifentanil TCI was also effective and safe in spontaneously breathing patients with severe acute hypoxemic respiratory failure, when FOB could not be performed under single topical anesthesia [21]*.*

We aimed at assessing the feasibility of using remifentanil TCI without adjunction of any other medication during FOB in the ICU. We also evaluated patients’ tolerance as well as operators’ and patients’ comfort during the procedure.

## Materials and methods

### Study design and population

This is a prospective non-interventional, observational, monocentric study. All consecutive patients requiring a FOB in our ICU as per treating physician were considered for inclusion between May 2014 and December 2015. If several FOBs were performed with remifentanil TCI on a patient, only the first was considered for inclusion. Thus, to avoid any memorization bias, a patient could only be included one time. Inclusion criteria required the patients to be conscious (Richmond Agitation Sedation Score (RASS) > -3) regardless of the use of mechanical ventilation [22]. Patients younger than 18 years, pregnant or breastfeeding women, comatose patients (Glasgow score < 8), with a contraindication to remifentanil, or unable to provide their consent were excluded. This study was conducted in accordance with the amended Declaration of Helsinki and was approved by the local ethics committee. All patients consented to participate.

### Protocol

Remifentanil was infused with a 2 ng/mL initial brain effect-site concentration (Cet). FOB started only when this target was reached. A topical anesthesia for the nose (2% lidocaine chlorhydrate gel), the oropharynx (5% lidocaine chlorhydrate spray) and the tracheobronchial tree (1% lidocaine chlorhydrate, 5 mL) was used as per department’s protocol.

Remifentanil target-controlled infusion (Base Primea, Fresenius®Kabi, Minto model) was then titrated by 0.5 ng/mL steps according to patient’s reactions with a maximum of 6 ng/mL of brain Cet. The procedure started when adequate sedation (Observer Assessment of Alertness and Sedation scale (OAA/S) score 2–3) was reached [23]. Potential side effect managements were protocolized and are specified in the Additional file 1. In case of patient discomfort or agitation, 0.25 mg/kg of propofol could be infused to allow achievement of the procedure in optimal conditions.

Clinical data were collected every 2 min during the procedure (respiratory rate, oxygen saturation, heart rate and blood pressure) and patient’s comfort was evaluated with three hetero-assessment scales: the Puchner scale [24], cough scale, and OAA/S scale (Table 1). Total dose of remifentanil, maximal Cet and infusion duration were collected at the end of the procedure. Respiratory parameters were reassessed 1 h and 24 h after the end of the procedure. We defined intensification of ventilatory support as an increase in the need of oxygen, or a rise in the level of respiratory care from oxygen therapy to non-invasive (NIV) or invasive ventilation in previously spontaneous breathing patients.

**Table 1:** Scores used for comfort, cough and sedation (by OAA/S and RASS scores)

Comfort and cough scale		2	3	4	5
Comfort*	No reaction	Slight grimacing	Heavy grimacing	Head or limb defending	–
Cough**	None	Slight	Moderate	Important	–

OAA/S scale***	1	2	3	4	5
Responsiveness speech	Does not respond to noxious stimuli	Responds only after mild prodding or shaking	Responds only after name called loudly and/or repeatedly	Lethargic response to name spoken in normal tone	Responds readily to name spoken in normal tone
Facial expression	–	–	Marked relaxation	Mild relaxation	Normal
Eyes	–	–	Glazed and marked ptosis	Glazed or mild ptosis	Clear, no ptosis

RASS score****	– 5	− 4	− 3	− 2	− 1
RASS	Unarousable	Deep sedation	Moderate sedation	Light sedation	Drowsy

^*^ Puchner et al. [23]

^**^ Chalumeau et al. [20]

^***^ OAA/S scale: Observer Assessment of Alertness/Sedation scale which evaluates the level of consciousness in patients sedated [22]

^****^ Richmond Agitation Sedation Score [21]

The operator rated his/her comfort in performing the procedure on a numeric scale going from 0 (“very uncomfortable conditions”) to 10 (“maximal comfort”) after the end of the FOB.

To avoid any judgement alteration due to residual effect of the drugs, patients’ experience assessment was performed 24 h after the procedure: pain and comfort were assessed with numeric scales going from 0 to 10. Tolerance, memorization and theoretical approval for repetition of the same procedure in similar conditions were assessed [25].

### Outcomes

The main outcome was the rate of procedure success defined as the achievement of the FOB with remifentanil TCI alone and no requirement for additional sedative drug.

The secondary objectives were the clinical tolerance of the FOB and sedation, in particular respiratory tolerance evaluated by the intensification of ventilatory support after the procedure, and the patient’s and the operator’s comfort during the procedure.

### Statistics

Continuous variables are reported as means ± SD or median (first, third quartiles), and categorical variables as count and proportion. Normality of the data distribution was visually assessed by means of histograms. Comparisons of proportions were made using Chi-square and Fisher exact tests. Continuous variables were compared using Student’s t tests or Wilcoxon rank sum test statistics. Numeric variables at two different time points were compared with paired t-test or Wilcoxon test. No assumptions were made for missing data, and we followed the Strengthening the Reporting of Observational Studies in Epidemiology (STROBE) recommendations [26]. Statistical analyses were done with R (version 3.5.1, http://cran.r-project.org). All P values were two-sided, and value less than 0.05 was considered statistically significant.

### Results

Between May 2014 and December 2015, 72 patients requiring a FOB were included. Forty percent were men, with a mean age of 57 ± 17 years and a mean Simplified Acute Physiological Score (SAPS2) of 33 ± 16. Main reasons for ICU admission were respiratory failure (69%), thoracic trauma or surgery (21%). Most patients were hypoxemic: 46% needed more than 8 L/min of oxygen, 21% received high-flow humidified oxygen therapy through nasal cannula (HFNC), 10% were treated with NIV. None required vasoactive drugs at the moment when the procedure was performed. Patients’ baseline characteristics are shown in Table 2.

**Table 2:** Demographic and baseline characteristics

Age, mean ± SD, years	57.4 ± 17.4
Male, *n* (%)	29 (40)
BMI, mean ± SD, kg/m^2^	25 ± 6
SAPS2, mean ± SD	33 ± 16
Comorbidities, *n* (%)	
Active smoking	35 (49)
COPD	23 (32)
Other pulmonary disease	13 (18)
Chronic heart disease	4 (6)
Immunosuppression	30 (42)
Main cause of ICU admission, *n* (%)	
Respiratory failure	34 (48)
Pneumonia	20 (28)
Acute interstitial pneumonia	7 (10)
Acute chest syndrome	4 (6)
Other	3 (4)
Hemoptysis	19 (26)
Thoracic surgery/traumatism	11 (15)
Abdominal surgery	1 (1)
Extra-pulmonary sepsis	2 (3)
Other	5 (7)
Pre-bronchoscopic respiratory parameters	
SpO_2_, median [IQR], %	97 [94.8–99.2]
RR, mean ± SD, per min	27 ± 9
PaO_2_ < 60 mmHg, *n (%)*	17 (24)
RR > 30 cycles per min, *n* (%)	34 (47)
Respiratory support, *n* (%)	
Oxygen therapy > 8L/min	30 (42)
HFNC	15 (21)
NIV	7 (10)
Invasive mechanical ventilation	4 (6)
Pre-bronchoscopic hemodynamic parameters	
MAP, median [IQR], mmHg	93 [83–101]
HR, mean ± SD, beat per min	99 ± 21

BMI: body mass index, SAPS2: Simplified Acute Physiologic Score, COPD: chronic obstructive pulmonary disease, SpO_2_: oxygen saturation by pulse oximetry, RR: respiratory rate, PO_2_: arterial partial pressure of oxygen, HFNC: high-flow humidified oxygen therapy through nasal cannula, NIV: non-invasive ventilation, MAP: mean arterial pressure, HR: heart rate

A total of 72 procedures were assessed in 72 different patients. A concomitant broncho-alveolar lavage (BAL) or bronchial biopsies were performed in 22% and 4.2% of them, respectively. The main indications for FOB were worsening of respiratory failure, etiological diagnosis inquiry or hemoptysis (*n* = 29, 22 and 18, respectively). Four procedures (6%) were performed in intubated patients. The median [IQR] duration of remifentanil infusion was 22 [15–28] min for a median procedure duration of 11 [8–19] min. The total dose of remifentanil was 252 [164–343] µg, with a mean maximal Cet of 4.4 ± 1.2 ng/mL.

Sixty-nine procedures (96%) were successfully performed without any additional sedative drug requirement (primary outcome). No severe adverse event occurred, and no reversal agent was ever used (naloxone). Physiological parameters are showed in Table 3.

**Table 3:** Physiological parameters at baseline, during the procedure and after 1 h (median [IQR])

	Baseline	Minimal per-procedure	Maximal per-procedure	After 1 h
SpO_2_ (%)	97 [94–99]	94 [92–97]*	99 [97–100]*	97 [95–99] *p* = 0.244
RR (cycles/min)	27 [20–31]	18 [12–22]*	30 [24–36]*	23 [20–28] *p* = 0.004
HR (beats/min)	100 [83–111]	95 [77–107]*	106 [93–124]*	94 [83–106] *p* = 0.026
SAP (mmHg)	139 [118–149]	126 [110–137]*	151 [139–168]*	130 [118–137] *p* = 0.052
MAP (mmHg)	93 [83–101]	87 [80–95]*	104 [93–113]*	89 [81–98]*

SpO_2_: oxygen saturation by pulse oximetry, RR: respiratory rate, HR: heart rate, SAP: systolic arterial pressure, MAP: mean arterial pressure. Parameters compared with baseline with paired Wilcoxon test

^*^p < 0.001

Oxygen levels were increased for the procedure in patients treated with standard oxygen therapy (6 [4;9] vs. 4 [2;6] L/min, *p* = 0.013) as well as FiO_2_ in patients receiving NIV, HFNC or invasive mechanical ventilation (1 [0.60;1] vs. 0.6 [0.4;1], *p* = 0.008).

The minimal per-procedure oxygen saturation was significantly lower than that at baseline (94% [92–97] vs. 97% [94–99], *p* < 0.001), but SpO_2_ 1 h after the procedure did not differ from baseline SpO_2_ (*p* = 0.244).

All but one patient had the same type of ventilation support before and during the procedure. This patient was treated with 12L/min oxygen through non-rebreather mask before the procedure, and treatment was increased to high-flow humidified oxygen therapy through nasal cannula to secure the procedure.

The ventilatory support was increased in 13 (18%) patients within 24 h after the procedure, most of them for worsening hypoxemia (*n* = 10; one patient required invasive mechanical ventilation, another needed NIV, the remaining 8 patients required higher oxygen levels). Three other patients required invasive mechanical ventilation for emergent surgery unrelated to the FOB procedure. Conversely, 41 patients (57%) needed less oxygen within 24 h after the procedure. There was no difference of remifentanil dose administered between the 13 patients who presented respiratory failure worsening and the 59 other patients (264 [147–384] vs 249 [169–348] µg, *p* = 0.838).

**Fig. 1:**
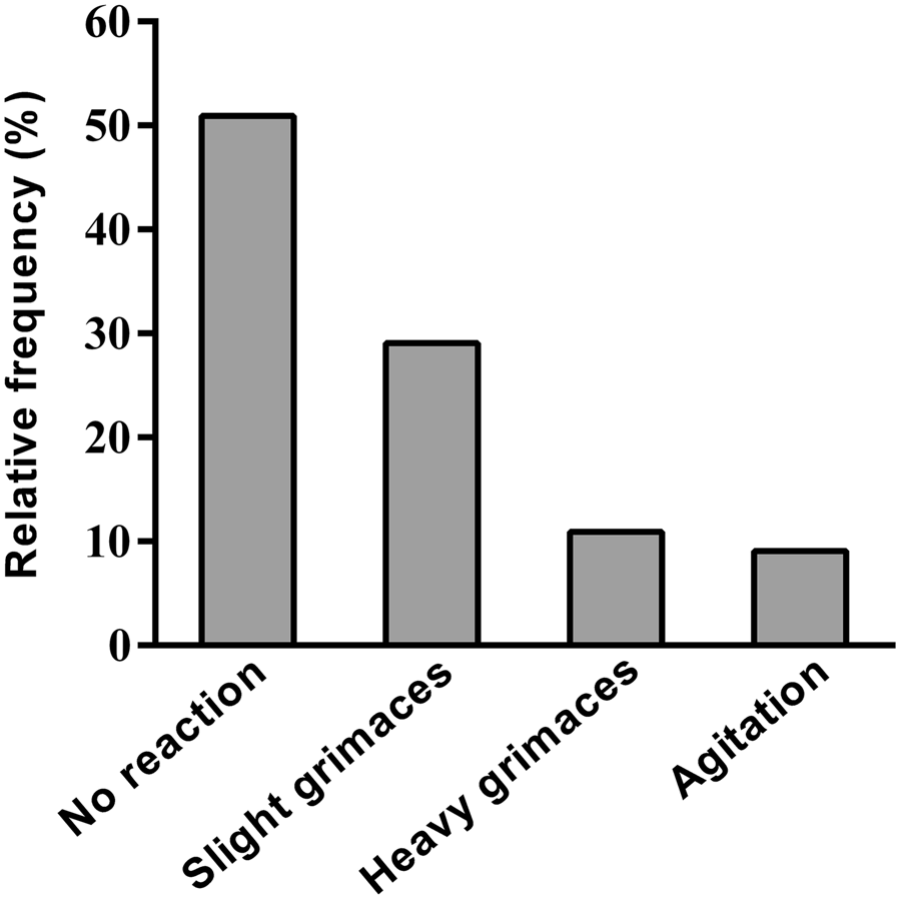
Evaluation of the patient's comfort during the procedure using Puchner score (obtained for 55 patients)

During the FOB, 30.6% of the patients presented no cough, 59.7% a moderate cough, and 9.7% an important cough. The evaluation by Puchner score was performed in 55 patients, among whom 51% showed no reaction. Slight grimaces, heavy grimacing, and agitation were noticed in 29.1%, 10.9% and 1.8% of patients, respectively (Fig. 1).

Subjective perception of the procedure was assessed at 24 h in 50 patients (69.4%). The great majority of them (98%) could remember at least a part of the procedure, 78% felt “comfortable” or “very comfortable”, 96% suffered “no pain” or a “moderate pain” and 85.4% declared they would accept to repeat the procedure under the same conditions if needed (Table 4). Fifty-one percent of the physicians reported on their comfort performing the procedure and the median [IQR] score was 9 [8–10].

**Table 4:** Evaluation of patient’s comfort and perceptions (assessed in 50 patients). Results are presented as *n* (%)

Memory, n (%)*	
I remember everything	27 (55.1)
I remember the most part of the procedure	14 (28.6)
I remember few things	7 (14.3)
I don’t remember anything	1 (2.0)
*Comfort: “During the procedure, I felt…”,* *n* *(%)*	
Very comfortable	9 (18.0)
Comfortable	30 (60.0)
Uncomfortable	8 (16.0)
Very uncomfortable	2 (4.0)
I can’t tell	1 (2.0)
*Pain: “During the procedure, I had…”,* *n (%)*	
No pain	32 (64.0)
Moderate/bearable pain	16 (32.0)
Unbearable pain	1 (2.0)
I can’t tell	1 (2.0)
*Tolerance: “I would agree to to undergo the same procedure in the same conditions”, n* (%)**	
Certainly yes	20 (41.7)
Likely yes	21 (43.8)
Likely not	5 (10.4)
Certainly not	1 (2.1)
I can't tell	1 (2.1)

The patients were asked about their perceptions 24 h after the procedure

^*^Assessed in 49 patients

^**^Assessed in 48 patients

### Discussion

This study showed that the use of remifentanil TCI for FOB was feasible, safe and enabled comfortable examination settings for the patients as well as for the physicians in charge of the procedure. This analgesic protocol was well tolerated despite being performed in ICU patients with respiratory failure.

Patients developed a transient and reversible desaturation and physicians increased the levels of oxygen or FiO_2_ for the procedure, likely anticipating procedure induced hypoxemia expected in this fragile patient population. Only one patient had a change of oxygen delivery device for the procedure, escalating from non-rebreather mask (12L/min) to HFNC. In this patient, the broncho-alveolar lavage allowed diagnosis of pneumocystis pneumonia and in this context, the increment of ventilation support would have occurred with or without the procedure. FOB may lead to transient hypoventilation and drop of oxygenation [27], especially during BAL. In a prospective multicenter study, bronchoscopy performed in critically ill hypoxemic patients was associated with an increase in ventilatory support [28]; immunosuppression and COPD were associated with the need for intubation within the 24 h after the procedure. In our study, patients with immunosuppression and COPD represented, respectively, 42% and 32% of the population. However, only one of them was intubated after the FOB for an emergent surgery which was unrelated to the procedure. In this population, FOB was indicated to diagnose or treat pulmonary conditions likely influencing the levels of oxygenation and some patients’ respiratory conditions may have declined due to the natural course of their underlying disease or condition (pneumonia, hemoptysis, thoracic surgery).

None of the usual complications of opioids such as severe bradypnea, thoracic rigidity or significative bradycardia occurred, and no reversal agent was needed in our study [29–33]. It is comforting that these most feared complications did not happen in a population of 72 hypoxemic patients. As remifentanil is a very potent and short-acting opioid, we assume that TCI administration allowing a quick equilibration of plasmatic and tissues’ concentrations is a safe way of delivering this drug. Recently, Rezaiguia et al. performed FOB under remifentanil TCI sedation in a series on 39 hypoxemic patients. The authors reported an increase in ventilatory support after the procedure in 23% of them [21]. Noteworthy, all these patients had a recent thoracic surgery and a previous failure of FOB under topical anesthesia because of discomfort, agitation or respiratory failure. In a multicenter cohort of 169 spontaneous breathing ICU patients with acute respiratory failure and no information regarding the sedation use, 35% of the awake FOB leaded to an increase in ventilatory support [28]. In our study, better tolerance of the procedure and the lower rate of ventilatory support increase could be explained by the pharmacological effects of remifentanil and its delivery by a TCI syringe, thus allowing precise titration, personalized treatment, and preventing side-effects as well as insufficient sedation.

Patients whose experience was assessed mainly rated it as “comfortable” or “very comfortable”. This was consistent with physicians’ perceptions who mostly appreciated examination conditions. Therefore, our results, suggest that we may improve patient’s experience and operator’s comfort without increasing the risk of the complications.

Awake FOB is known to be unpleasant and an important source of pain and anxiety [3, 4, 34]. Respiratory tract stimulation causes cough leading to patient’s discomfort and technical challenge for the physician.

Moreover, FOB can cause dyspnea and desaturation in patients who are already at risk due to their underlying pathology. Studies comparing remifentanil TCI with propofol TCI found that intubation conditions as well as patient comfort were better with remifentanil [35, 36]. Remifentanil might be an appropriate drug for respiratory tract procedures because it modulates the perception of dyspnea [37–39], but also due to its strong analgesic properties [16, 17]. FOB is painful and other drugs usually administered for this procedure do not have such analgesics power [40–42]. Remifentanil provides a better satisfaction than a procedure without any sedation and is efficient on different components of suffering: pain, dyspnea and anxiety. Morphine and midazolam titrations are easy to implement, but both have longer onsets and offsets making them less adjustable. Ryu et al. compared a vigil sedation with propofol–dexmedetomidine, and propofol–remifentanil to perform vigil FOB [43]. In the dexmedetomidine group, patients experienced fewer transient desaturations, but they also reported less comfort and satisfaction. In 2011, Clouzeau et al. performed BAL under awake sedation with propofol in 23 patients with acute respiratory failure under NIV. Respiratory status one hour after the procedure was not different from baseline. Three patients (17%) were intubated 24 h after the procedure and mean arterial pressure was transiently lower under sedation, but stayed in acceptable levels [44]. As opposed to our study, all procedures were performed under NIV which was maintained for at least 1 h after FOB and authors used propofol. Propofol used for vigil FOB in a small series of 18 patients with pneumonia showed improved patient satisfaction, with reduced cough, pain and sensation of asphyxiation as compared to procedure without sedation [25]. Propofol and remifentanil are both suitable molecules for the sedation of short procedures as both have the rapid onset/short-acting effect and both have good results on the control of procedural pain. However, propofol induces more amnesia, which comes with hemodynamic repercussions by sympatholysis and has a larger impact on the oropharyngeal tonus with an increased risk of obstructive apnea [35]. These expected side-effects can be partially avoided by a close management of its administration, or a combined sedation, for example with ketamine [45].

Alleviating acute, chronic or procedural dyspnea, considered as unpleasant and harmful as pain, should be a daily goal for the physicians [46]. Though little has been reported on the painfulness of FOB, it is undoubtedly an unpleasant procedure [4, 47], particularly in patients from this cohort who suffered from independent sources of anxiety such as dyspnea or hemoptysis. Considering the negative impact of stress on the perception of pain [6–10, 48], assessing and relieving patients’ stress and pain levels becomes a priority. Hence, current guidelines already recommend the use of sedation to perform this procedure [49]. Several studies have also demonstrated the benefit of a conscious sedation in ambulatory patients [40, 50]. Improving patient’s and physician’s comfort during the procedure also seems to be important in critically ill hypoxemic patients.

We chose remifentanil for its appropriate pharmacological properties combining rapid onset, easy adjustment and short acting. Patients required high levels of Cet (in the range of those needed for a surgical incision) to control pain and discomfort and this is consistent with a recent study on FOB in ICU patients suffering from acute respiratory distress syndrome in which Cet exceeded 4 ng/mL [21]. Several hypotheses could explain this phenomenon. First, the procedural pain might have been underestimated. The need for high levels of Cet could reflect higher levels of suffering during the procedure, and could also translate the impact of baseline pain on procedural pain (i.e., greater suffering in ICU patients due to a higher baseline) [1]. Second, Cet was adapted depending on patient’s reactions, cough, movements and grimaces, as in most cases patients could not talk during the procedure. Thus, we could have incremented remifentanil doses to inhibit cough reflex during the procedure, without consequences as no severe complications and no signs of overdose occurred. No perfect tool exists, and hetero-assessment is a difficult task. In this study, we combined different auto- and hetero-assessment questionnaires answered at different times to limit the risk of judgment error.

Our study’s main limitation is the monocentric and non-controlled design. Still, remifentanil TCI for fiberoptic bronchoscopy remains relevant even for center with no or limited exposure to this technique: when it was implemented in 2012, only one physician was familiar to this technique and introduced it to the rest of the team. The whole team including most physicians with a respirologist background (used to perform this procedure with topical anesthesia for decades) quickly adopted remifentanil TCI sedation and became routine standard of care in our center. We thus considered not ethical to select a control group not receiving optimal pain medication.

In comparison with other ICU cohorts, our population had a stable hemodynamic status and did not require vasoactive agents. We did not show significant variations in hemodynamics status, but it may be interesting to test the same procedure on hemodynamically unstable patients. However, as remifentanil has been shown to be well tolerated in previous studies, we expect similar results.

Assessments of perceptions about the procedure were not completed for all patients and physicians. Two-thirds of the patients were asked about their comfort after 24 h, and half of the physicians assessed their conditions of examination. We think this is missing data at random due to organizational setting (missed 24-h follow-up time when it happened during weekends or after a staff change), and there was no specific difference between patients that were assessed and the others.

Finally, in the future, we should consider a longer follow-up to evaluate the impact of our pain management protocol on patients’ feelings after ICU and hospital discharge. The promising results of this study should be integrated in a global approach of patients’ comfort in ICU.

### Conclusion

Remifentanil TCI is feasible and safe to provide comfort for patients requiring awake flexible fiberoptic bronchoscopy in the ICU. This is a readily available tool that could appropriately alleviate procedural pain and improve the patient’s experience as well as the operator’s comfort.

## Supplementary Information

**Additional file 1.** Management protocols for procedural and sedation complications.

## Data Availability

The dataset used and analyzed during the current study are available from the corresponding author on reasonable request.

## Abbreviations

BAL: Broncho-alveolar lavage
BMI: Body mass index
Cet: Brain effect-site concentration
COPD: Chronic obstructive pulmonary disease
FOB: Fiberoptic bronchoscopy
HFNC: High-flow humidified oxygen therapy through nasal cannula
HR: Heart rate
ICU: Intensive care unit
MAP: Mean arterial pressure
NIV: Non-invasive ventilation
OAA/S: Observer Assessment of Alertness and Sedation scale
PO_2_: Arterial partial pressure of oxygen
RASS: Richmond Agitation Sedation Score
RR: Respiratory rate
SAP: Systolic arterial pressure
SAPS2: Simplified Acute Physiological Score
SpO_2_: Oxygen saturation by pulse oximetry
TCI: Target-controlled infusion
